# Do institutions matter for citizens’ health status? Empirical evidence from Italy

**DOI:** 10.1007/s10198-024-01689-9

**Published:** 2024-05-09

**Authors:** M. Alessandra Antonelli, Giorgia Marini

**Affiliations:** Department of Legal and Economic Studies, Sapienza University of Rome, Rome, Italy

**Keywords:** Life expectancy, Mortality, Morbidity, Health status, Institutional quality, Italian regions, H75, I18, I10, P48

## Abstract

This paper investigates the role of institutional quality in explaining cross-regional variation in population health status in Italy. We first introduce a composite Regional Health Status Indicator summarizing life expectancy, mortality and morbidity data. Then, we study the empirical relationship between this indicator and a set of socioeconomic, health system and institutional controls at the Italian regional level over the period of 2011–2019. We find that institutional quality is a driver of population health. Furthermore, well-functioning local institutions and regions compliant with national standards in terms of public healthcare services (Essential Levels of Care) make socioeconomic context no longer relevant for population health, potentially reducing inequalities.

## Introduction

The purpose of this paper is to empirically explore the relationship between the quality of institutions and population health at Italian regional level. The issue of institutions has recently drawn considerable attention in the health economics literature, as it acknowledges the fact that health outcomes depend not only on socioeconomic, political, and cultural factors but also on the capacity of institutions to offer public services efficiently, effectively and on time [1]. For this reason, it is obvious that, for example, universal health coverage—free at the point of use—can be established by law, but if the healthcare system is not adequately financed, governed and monitored, the effect on the health of the population might be detrimental. Good health system governance also requires civil society participation and government transparency and accountability [37].

These considerations lead to the issue of extending the traditional set of health explanatory variables to also include institutional quality elements to shape and develop an intersectional framework in which these two orthogonal sets of determinants are instead treated as two complementary determinants [47]. Within this framework, Knowles and Owen [61] study the role of institutional variables in explaining cross-country variations in life expectancy in a sample of 73 high- and low-income countries. Using indicators related to both formal institutions (ruled by law and regulation) and social capital (ruled by conventions, social norms and codes of behaviours), they show that an improvement in institutions has a statistically and quantitatively significant positive effect on life expectancy. Similarly, Hadipour et al. [48], using a panel dataset from 158 high- and low-income countries between 2001 and 2020, find that institutional quality has a positive impact on life expectancy and a negative impact on infant mortality rates. Narrowing to the EU context, Sharma et al. [83] find a positive association between the quality of institutions and infant mortality rate and life expectancy at birth. This kind of relationship is also confirmed by Holmberg and Rothstein [50], whose analysis highlights a positive correlation between several variables of government quality (rule of law, corruption and government effectiveness) and life expectancy and a negative correlation with infant and maternal mortality rates. Narrowing the analysis of institutional quality to the specific aspect of corruption, several studies point out that low healthcare system performance in terms of efficiency, effectiveness and equity can be found in a highly corrupted context [46], with a consequent adverse impact on health outcomes such as life expectancy and mental health [1], general mortality and infant mortality [49].

Our contribution can be collocated in this strand of literature, introducing some elements of originality.

First, compared to the existing studies, our approach adopts a new measure of health status represented by a multidimensional composite indicator. This allows us to account for various dimensions of health, shifting the analysis to a more general level.

Second, we have identified only very few studies validating a significant relation between quality of institutions and observed or self-perceived physical health of people in the Italian context [42]. Therefore, this paper contributes to the literature assessing the relationship between institutional quality and a broad indicator of physical health in Italy, where healthcare is managed by region but subject to guidelines of the central government setting national targets both in terms of healthcare service provision and budget accountability.

The aim of our paper is thus to disentangle the socioeconomic context characteristics, the regional healthcare system resources (staff and beds) and the policy/institutional explanatory variables (compliance with the national standards and quality of local institutions) and to investigate how Italian regional population health is related to these macro-measures.

Many empirical analyses point out a positive association between socioeconomic factors, variously defined (income, education, wealth) and a wide range of health indicators (such as, for example, mortality, life expectancy and morbidity). This thesis has been supported by different perspectives of analysis. Most studies focus on the relationship between individual socioeconomic standing and health indicators within single countries [20, 60, 62, 63, 85, 88, 90]. Fewer studies have examined the relationship across countries. In this framework, a set of research is microdata-based and analyses the relationship between individual socioeconomic variables and health showing that people with more economic resources (typically income and wealth) tend to be healthier than people with less resources in a comparative cross-national perspective [7, 13, 69, 82]. A further body of studies is represented by cross-national or cross-regional macro-level ecological analysis investigating the relationship between structural characteristics of territorial areas (nations, macro-areas, regions, counties) and health indicators. Such socio-ecological approach basically highlights that better health characterizes societies with better socioeconomic context and more egalitarian distribution of income [8, 24, 78].

Our analysis is carried out at Italian regional level with macro-variables, and it draws in the last research approach.

We use a cross-sectional sample of 21 Italian territorial units (19 regions plus 2 autonomous provinces, Bozen and Trento)^1^ observed for 9 years (2011–2019) to test the empirical relationship between health population and institutional quality at regional level also controlling for local socioeconomic variables and healthcare service features. The regional-level analysis is particularly interesting for Italy for at least two reasons: first, Italy has a fairly pronounced disparity in socioeconomic context (in terms of income, income inequality, education) and performance of institutions between regions and macro-areas; second, while some guidelines and targets of health policy are determined by the central government, the management of healthcare is entrusted to the regions.

We find that at Italian regional level, higher institutional quality is associated with higher health status of the population. Moreover, regional socioeconomic factors appear not to be a relevant driver for overall population health when local institutions are well-functioning and regions present a higher level of compliance with national standards in terms of public healthcare services.

The paper is organized as follows. In Sect. Institutions and health: an overview, we provide an overview of the literature on the relationship between institutions and health and state our research hypotheses. In Sect. Assessing health: The Regional Health Status Indicator, we introduce the Regional Health Status Indicator and its computation methodology. Section Data and variables describes the data and the variables used in the empirical analysis. Section Empirical strategy introduces the empirical strategy and Sect. Empirical results discusses the results. Finally, Sect. Conclusions concludes the paper.

## Institutions and health: an overview

A long tradition in the socioeconomics literature focuses on the positive relationship between socioeconomic status and health. From this perspective, population health status is related to socioeconomic dimensions as income, wealth, education, occupation, gender, and ethnicity such that people lower in the social hierarchy have poorer health than people higher in the social hierarchy (for a review [30, 68]). However, several studies adopt a wider perspective of analysis by examining the relationship between health and institutions [47, 61]. One of the most important contributions on the theory of institutions comes from Douglas North who defines the institutions as “the humanly devised constraints that structure political, economic and social interaction” [75] p.97). They consist of both formal and informal rules corresponding to formal and informal institutions. Formal institutions include the written constitution, laws, policies, rights and regulations enforced by official authorities and influencing individual well-being [18],informal institutions are usually unwritten norms of behaviour, codes of conduct, customs, conventions that shape thought and behaviour[17, 66].^2^ Formal institutions are easier to identify because they are based on codified rules that define a framework within which human interaction takes place, informal institutions are typically not codified and harder to observe and classify.^3^

Given the heterogeneity of institutions operating within a society, the interaction between institutions and health is a complex issue that has been addressed by researchers and policy makers not only at national but also at international [48] and European level [83].

Against this background, a field of socioeconomic studies consider the relationship between the health and the welfare state regimes by conceiving the latter as a set of formal institutions- or formal rules-governing the distribution of resources and opportunities among citizens [56, 71]. Health institutions are included in this general scope and concern the formal rules affecting the opportunities of care as the establishment of a minimum standard of care for citizens, the access to the healthcare services and the extent of co-payment for treatments, the location and the organization of health facilities etc.^4^ Beckfield et al. [14] propose a theoretical framework that emphasizes the role of the welfare state institutions in distributing population health. They argue that welfare states can stratify health through two macro-channels.^5^ The first one concerns health institutions. They present different features across welfare state regimes (rules concerning the financing of healthcare and their decommodification^6^ degree, a minimum level of prevention and healthcare, the spatial localization of health facilities, the regulation of private providers etc.), thus distributing health differently. A second way is related to the distribution policies for income and other valuable goods (employment security, minimum wage, wage replacement rate, pensions, working security, housing, education) that vary by welfare state regime and affect the social determinants of health.

In a different perspective of analysis and looking at population health level indicators, Beckfield and Bambra [15] implement a time-series cross-section analysis from 1970 to 2010 for the US and 17 other high-income countries to assess the association between generosity of welfare state institutions (for unemployment insurance, sickness benefits, and pensions) and life expectancy. Their analysis provides evidence of a strong role for social policy shortcomings in explaining the negative gap of life expectancy for US with respect to other rich democracies. In the same strand of research, Jacques and Noël [53] observe a negative relationship between welfare state decommodification and the age-standardized death rate for 21 OECD countries from 1971 to 2010. Their findings confirm that social programmes providing better social protection and making individuals less dependent form the market, are associated to healthier lives.

Moving on to consider other formal institutions, law and the justice sector also play an important role for citizens’ health. Environmental protecting laws, as well as the norms prohibiting the marketing of harmful foods impact on the health of individuals. In a broader perspective, Dingake [37] highlights that only a well-functioning rule of law makes effective the health right and the healthcare access. Access to justice, whether to courts, alternative dispute resolution mechanisms, or traditional justice systems can improve access to healthcare services, in particular for marginalized populations [37] p. 296). Well-functioning legal systems also provide control over contextual factors such as corruption, bureaucratic inefficiency, protection from crime and government accountability, that the literature has identified as significant for population health [61]. Using a cross-sectional sample of 185 countries, Achim et al. [1] show—for the period 2005–2017—that the level of corruption significantly affects physical health (measured as life expectancy and mortality) as well as mental health. Corruption determines the misappropriation of funds and medical equipment, making access to health services more difficult, reducing their effectiveness and leading to worse health outcomes [46]. Robinson and Keithley [79] provide evidence that widespread crime directly affects physical and psychological health of victims but also leads to a substantial increase of the medical care demand, placing additional pressure on services and diverting resources from other patients with potential negative effects in terms of population health outcomes.

The connection between health and the institutional framework also concerns the in*formal institutions*. Informal institutions are basically identified with social capital which is considered a byproduct of social organization. As a multidimensional concept, social capital includes several dimensions: social and civic participation, political participation, trust relationships, perceived social support, sense of belonging. High levels of social capital have been reported to be associated with better health and a lower risk of mortality [51, 58, 59, 80]. The idea is that social capital contributes to mental well-being through a trusting environment or through the benefits of socializing,it also improves physical health through the diffusion of information on the effectiveness of healthcare services and on health behaviours, promoting the mutual assistance, the sense of responsibility and thus reducing health-risky behaviours [43].

Nieminen et al. [72] analyses the associations between individual-level social capital and all-cause mortality among working-age and ageing people in Finland for the period 2000–2009, also controlling for socio-demographic, behavioural and biological factors. They find that the mortality rate was smaller among people with higher social participation activities compared with those socially inactive. Fiorillo and Sabatini [42] provide an empirical assessment of the causal relationship between social capital and health in Italy, finding that individual structural social capital, as measured by the frequency of meetings with friends, is strongly and positively correlated with self-perceived health. The same results characterize the analysis of Yuan et al. [91] carried out for China. Using cross-sectional data from the China Family Panel Studies 2016, the authors find that social relationships (measured by gift income) and organization membership have positive effects on self-relate health, from a micro-perspective.

In this variegated strand of literature, our paper investigates, at regional level, the empirical relationship between a broad indicator of institutional quality, including elements of formal and informal institutions, and overall population health, also controlling for socioeconomic and healthcare system characteristics.

In particular, we state the following hypotheses:

- Hp1: At regional level, higher institutional quality is associated with higher health status of the population.
- Hp2: Regional socioeconomic factors do not affect overall population health when local institutions are well-functioning and regions present a higher level of compliance with national standards in terms of public healthcare services (i.e., prevention, use of medical care, vaccinations and other health treatments etc.).

## Assessing health: the regional health status indicator

Our investigation into the relationship between institutional quality and health is based on a multidimensional composite indicator summarizing several components affecting the health status of the population.^7^ We propose a Regional Health Status Indicator (RHSI) calculated at the local level for 21 territorial units for the period of 2011–2019. It is a combination of elements relating to both objective measures of health status and self-reported health.^8^

**Fig. 1:**
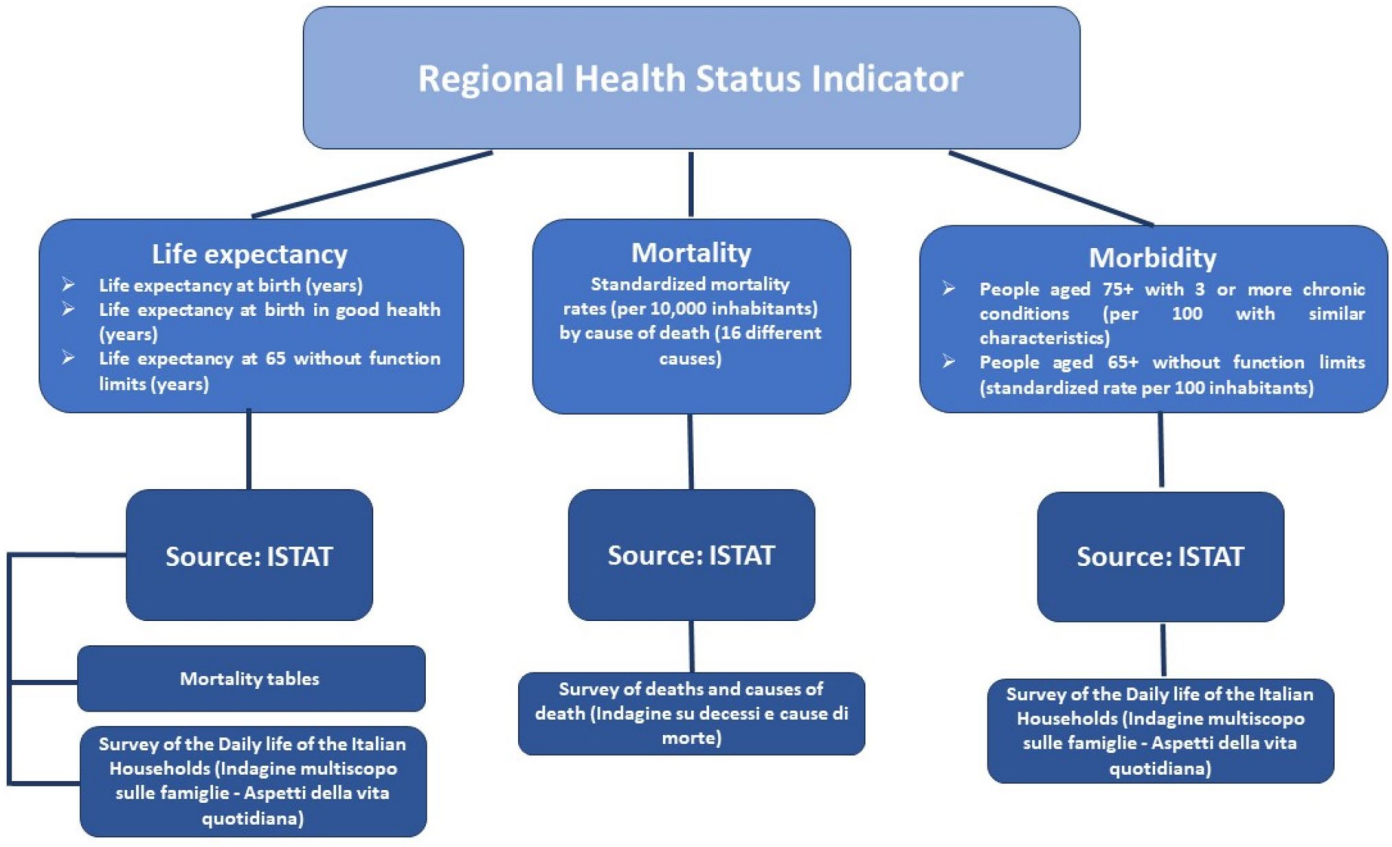
Domains, variables and data sources of the RHSI

The RHSI summarizes 21 elementary variables representing core aspects of both quantity and quality of life and health status concerning three domains: ‘life expectancy’, ‘mortality’ and ‘morbidity’. Figure 1 describes the variables included in each domain and the data sources.

Of the 21 variables, 4 are related to qualitative aspects of health status (‘Life expectancy at birth in good health’, ‘Life expectancy at 65 without functional limitations’, ‘People aged 75 + with three or more chronic conditions’ and ‘People aged 65 + without functional limitations’),^9^ and they are assessed with the self-reported measures of health included in the RHSI.

The remaining 17 variables are represented by the objective measures of health included in the RHSI: ‘Life expectancy at birth’ and ‘Standardized mortality rates’ that relate to 15 causes of clinically related deaths and 1 cause of death due to trauma, poisoning, homicide or suicide (classified as nonclinical deaths).^10^

The choice of the variables included in the RHSI is literature driven. The socioeconomic literature largely focuses on life expectancy [29, 63] and/or general mortality [54, 67, 70] treated, in some analyses, together with infant mortality [76, 84]. A more specialist (medical) literature relies on specific components of health status as multi-morbidity [28]. Most clinical studies place emphasis on reducing mortality rates and increasing life expectancy, both in terms of the length of life and the number of healthy life years [16, 86]. Fanshel and Bush [40] emphasize the need for indicators based on morbidity, while Segovia et al. [81] include chronic diseases functional limitations in their analysis of self-assessed health, among other variables.

From a computational perspective, we basically follow the methodology proposed by international organizations (United Nations,^11^ World Economic Forum^12^) for the computation of composite multidimensional socioeconomic indicators and applied by the economic literature [2, 5, 36]. First, as mortality rates and the indicator ‘People aged 75 + with three or more chronic conditions’ are naturally negatively oriented (i.e., the higher the indicator is, the worse the health condition is), we transform these two variables by taking their complementary value to have all components of the final RHSI positively oriented (i.e., such that higher scores are associated with better health status).^13^ After the transformation, the three domains can be newly defined as ‘Life expectancy’, ‘Survival’ and ‘No morbidity’. Then, to make comparison possible, each elementary variable is standardized by its national average:

1 $${x}_{v,j, i,t}^{\prime}=\frac{{x}_{v,j,i,t}}{{\overline{x} }_{v,j,t}}$$

where ${x}_{v,j,i,t}$ represents the value of elementary variable ${x}_{v}$ (*v* = *1, …, 21*) in domain *j* (*j* = *1, …, 3*) for region *i* (*i* = *1, …, 21*) at time *t* (*t* = *2011, …, 2019*).

Finally, the last step of the computation is the aggregation of the variables and domains. As in Di Bella et al. [36], we apply the following aggregation rules:

unweighted arithmetic mean of the ${x}_{v,j, i,t}^{\prime}$ variables within each domain *j*:

2 $${I}_{j,i,t}=\frac{{\sum }_{v=1}^{n}{x}_{v,j, i,t}^{\prime}}{n}$$

where ${I}_{j,i,t}$ is the synthetic measure of domain *j (j* = *life_exp*, *surv*, *no_morb*) for region *i* at time* t* and *n* represents the number of variables included in each domain;

unweighted geometric mean^14^ of the ${I}_{j,i,t}$ measures in the final ${RSHI}_{i,t}$ for region *i* at time* t*:

3 $${RSHI}_{i,t}={{(I}_{life\_exp,i,t}\cdot {I}_{surv,i,t}\cdot{I}_{no\_morb,i,t})}^\frac{1}{3}$$

under the hypothesis of assigning equal weights to the three components (${I}_{life\_exp,i,t}$, ${I}_{surv,i,t}$ and ${I}_{no\_morb,i,t}$) of the final ${RSHI}_{i,t}.$ Table 5 in Appendix A provides the RHSI values for 2011–2019.

## Data and variables

Our data are longitudinal, available annually for a period of 9 years (2011–2019) and for 21 territorial units with a total of 189 observations.

The main source of our data is ISTAT through the operating system Health for All-Italia, the data warehouse (http://dati.istat.it/) and the *Benessere Equo e Sostenibile* (BES) project.^15^ Other sources are the Ministry of Health and the Institutional Quality Index (IQI) dataset by Nifo and Vecchione [73], updated up to 2019.^16^

A description of the variables used in the empirical analysis is reported in Table 1.

**Table 1:** Variables description

Variable	Description	Source	Unit
Dependent variable			
RHSI	Composite indicator measuring the health of the population	ISTAT (derived)	Index
Socioeconomic factors			
Income	Per capita gross disposable income	ISTAT-BES dataset https://www.istat.it/it/benessere-e-sostenibilità/la-misurazione-del-benessere-(bes)/gli-indicatori-del-bes	Euro (current prices)
Education	People aged 25–64 who have completed at least upper secondary education (qualification not lower than ISCED level 3) out of the total number of individuals aged 25–64	ISTAT-BES dataset https://www.istat.it/it/benessere-e-sostenibilità/la-misurazione-del-benessere-(bes)/gli-indicatori-del-bes	Percentage
Unemployment	Unemployed individuals aged 15 + out of total number of individuals aged 15 +	ISTAT-Health for All database	Rate
Income inequality	Total equivalent income received by the 20% of the population with the highest income out of income received by the 20% of the population with the lowest income	ISTAT-BES dataset https://www.istat.it/it/benessere-e-sostenibilità/la-misurazione-del-benessere-(bes)/gli-indicatori-del-bes	Index
Drinking habits	People aged 14 + presenting at least one risky behaviour in alcohol consumption out of the total number of people aged 14 + (std rate per 100 inhabitants)^a^	ISTAT-BES dataset https://www.istat.it/it/benessere-e-sostenibilità/la-misurazione-del-benessere-(bes)/gli-indicatori-del-bes	Rate
Old population	Population aged 65 + out of total population	ISTAT-Health for All database	Percentage
Healthcare system variables			
Staff	Personnel (doctors and dentists, nursing staff, technical health personnel and rehabilitation staff) employed in public healthcare facilities^b^ per 10,000 inhabitants	ISTAT-Health for All database	Rate
Beds	Hospital beds for each type of activity (acute care, long-term care and rehabilitation) and facility (public and private accredited) per 10,000 inhabitants	ISTAT-Health for All database	Rate
PdR	Presence/subscription of a *Piano di Rientro* (Bail-out Plan)	Ministry of Health (derived)	Dummy
LEA	*Livelli Essenziali di Assistenza* (Essential Levels of Care) score	Ministry of Health	Number
Quality of institutions			
Government effectiveness	Measure of the endowment of social and economic structures in Italian regions and of the administrative capability of regional governments in terms of health policies, waste management and environment	IQI dataset https://sites.google.com/site/institutionalqualityindex/dataset	Index
Rule of law	Measure of perception concerning law enforcement both in terms of contract fulfilment, property rights, police forces, activities of the magistracy and crime levels	IQI dataset https://sites.google.com/site/institutionalqualityindex/dataset	Index
IQI	Institutional Quality Index measuring the overall quality of public institutions at local level (including elements of formal and informal institutions)	IQI dataset https://sites.google.com/site/institutionalqualityindex/dataset	Index
Instrumental variables			
Good transports	People aged 14 + satisfied about local public transports out of 100 people with the same characteristics	ISTAT	Percentage
Clean streets	Households living in areas where streets are clean out of 100 households with the same characteristics	ISTAT	Percentage

^a^See fotonote 20 and Appendix B for a more detailed description of this variable

^b^Public facilities include *Aziende Ospedaliere*, hospitals managed by local health authorities (ASLs), university hospitals, public and private scientific research and cure centres, classified or assimilated hospitals, residual psychiatric institutes, private institutes supervised by ASLs, and research centres

### Dependent variable

**Fig. 2:**
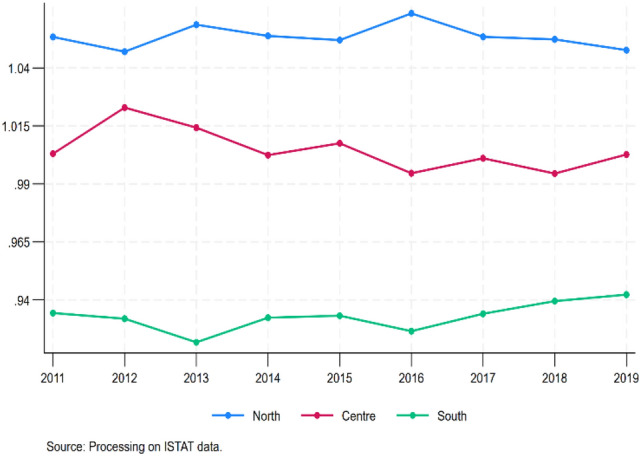
RHSI over time (2011–2019) by macro-area

Our dependent variable is the RHSI calculated at the regional level and introduced in Sect. Assessing health: The Regional Health Status Indicator. Figure 2 represents the RHSI trend over time (2011–2019) by macro-areas (northern, central and southern Italy). Territorial heterogeneity emerges across the macro-areas of the country, with the highest levels of the indicator for the northern regions and the lowest values for the southern regions. The central regions are in an intermediate position. However, the RHSI is rather constant over time. The standard deviation is approximately 0.006 for each of the macro-areas, meaning that data are clustered around the mean.

**Fig. 3:**
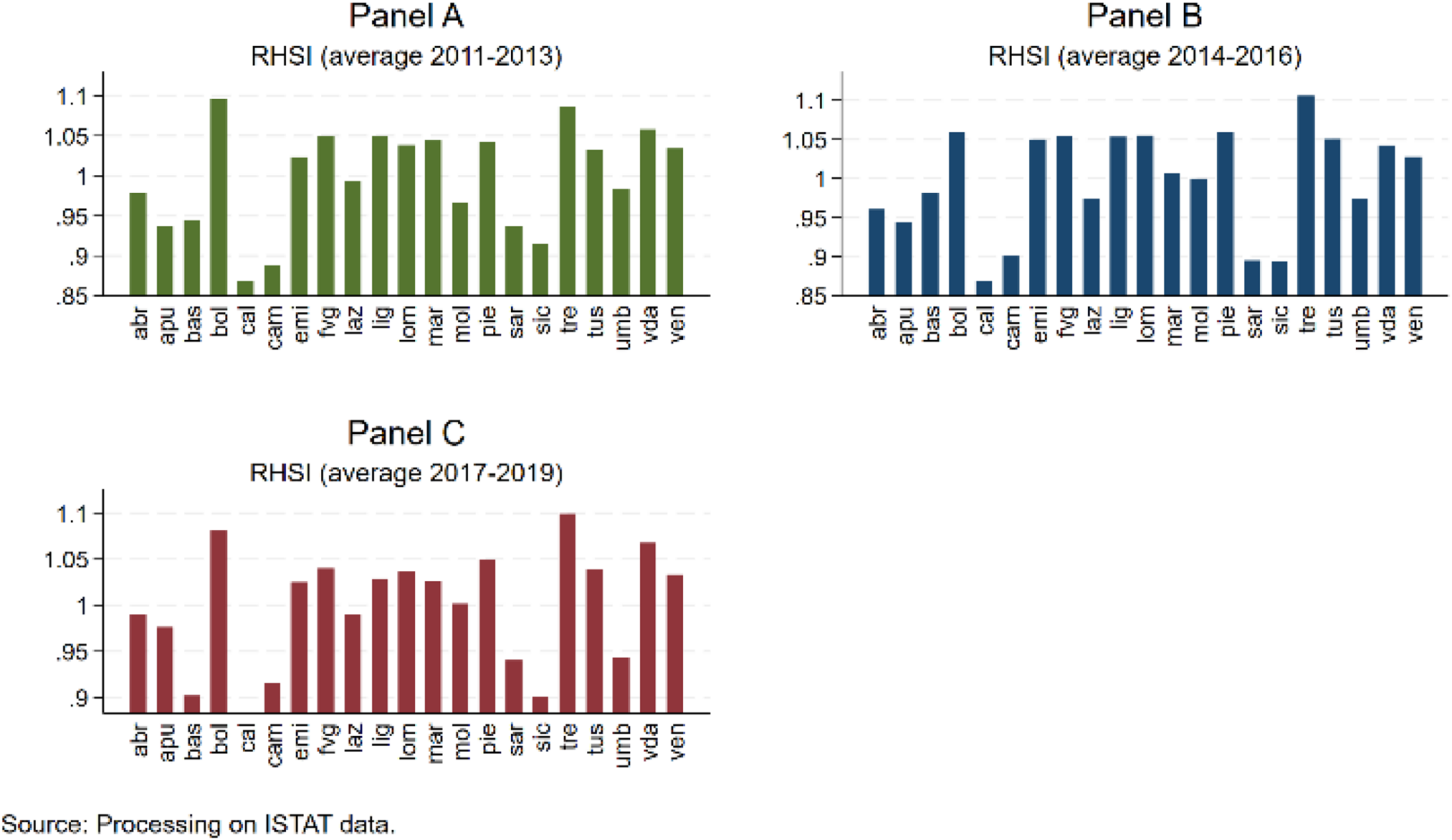
RHSI over time (2011–2013, 2014–2016, 2017–2019) by region

The territorial disaggregated analysis (Figs. 3, Panels A, B, C) also shows a certain heterogeneity among territories. In all three-year periods, the lowest value characterizes southern regions (Calabria, Campania and Sicily for 2011–2013; Calabria, Sardinia and Sicily for 2014–2016; Basilicata, Calabria and Sicily for 2017–2019), while the autonomous provinces of Trentino-Alto Adige record the highest level (Aosta Valley, Bozen and Trento for 2011–2013 and 2017–2019; Bozen, Piedmont and Trento for 2014–2016).

**Fig. 4:**
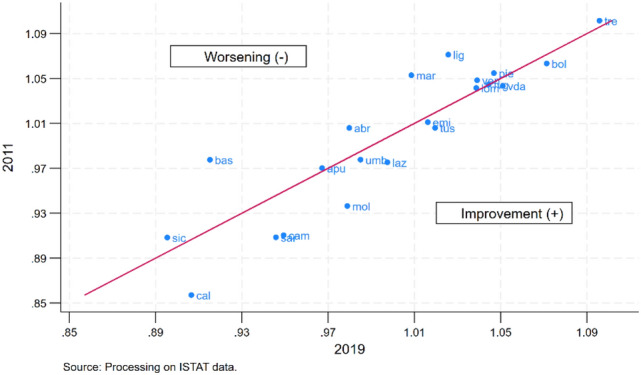
Regional comparison between RHSI for 2019 vs. 2011

Nonetheless, the comparison of the RHSI for 2011 and 2019 (Fig. 4) shows that among the regions facing an improvement in the indicator (those placed below and to the right of the diagonal), some southern regions, such as Campania, Sardinia and Calabria, recorded the largest increase.

### Explanatory variables

Explanatory variables used in the empirical analysis are divided into four categories: the socioeconomic component, the healthcare system, the quality of institutions and instrumental variables.

#### The socioeconomic component

The empirical evidence supports a positive association between good health outcomes and socioeconomic condition. The latter is an extended and multidimensional concept that is not only dependent on the availability of material resources (typically income and wealth) but also employment condition, cultural-behavioural factors (education, behavioural habits as drinking, smoking, sedentary lifestyle, diet) and other elements (gender, ethnicity) that give a better picture of social context also highlighting the possible exposure to social exclusion.^17^

Regarding the Italian context, Franzini and Giannoni [44] point out that populations living in regions with more poverty, more unemployment, more income inequality are more likely to report poor health. These results are supported by other empirical studies. In particular, Landi et al. [64] find evidence of a negative correlation between the socioeconomic context and waiting times for Italian National Healthcare Service (NHS) services. Their analysis suggests that, despite the Italian NHS statutory obligation to provide equal access according to needs to all Italian citizens, some population groups (low educated, less wealthy and low skill workers) are more vulnerable than others to experiencing excessive waiting times for healthcare services. Such a negative feature can induce patients with higher income and ability to pay to address their demand towards private healthcare services thus generating, in the Italian context, a pro-rich inequity in the access and use of healthcare services [26].

Against this background and to provide an in-depth picture of the socioeconomic regional Italian context, we consider a broad set of variables (described in Table 1) that, in our opinion, allows us to better delineate the economic characteristics of the regional context and to take into account some elements of social inclusion (or exclusion). To this end, in addition to variables widely used in the literature and accounting for the economic dimension (per capita income), the labour market performance (unemployment) and cultural features (education), we also control for the degree of differentiation of social status (income inequality),^18^ demographic characteristics (population over 65)^19^ and behavioural features (drinking habits).^20^

#### The healthcare system variables

A well-established part of the economic literature associates health outcomes with a production process carried out by the healthcare system through the use of productive factors such as capital and labour (among others, for Italy, [22, 31]).

From this perspective, we consider staff employed in public healthcare facilities and beds in public and private accredited facilities as explanatory variables in our analysis. Both can be considered proxies for labour and capital inputs financed by public resources. The variable ‘Staff’ includes both medical (doctors and dentists and nursing staff) and nonmedical staff (technical health personnel and rehabilitation staff) only employed in public healthcare facilities and paid by the public sector, while the variable ‘Beds’ refers to ordinary beds (i.e. acute care, long-term care and rehabilitation beds) employed in either public or private accredited facilities, as both types of beds are financed by the public sector in the Italian NHS.

In addition, we also consider some other elements introduced by the reforms that have interested the Italian NHS over the years since 1978.^21^ Originally a vertically integrated system of production and delivery, the Italian NHS has exhibited a progressive decentralization process over time. Regions were given greater power in the administration and organization of healthcare services in exchange for their acceptance of tighter budget constraints on healthcare expenditure. Many analyses have been devoted to assessing the effects of such reforms in terms of public healthcare expenditure [34], healthcare services provision [25] and citizens’ well-being [21, 77].

The 2001 constitutional reform introduced *Livelli Essenziali di Assistenza* (LEA), i.e. a set of public healthcare services to be provided and guaranteed to all citizens, either free of charge or on payment of a cost-sharing fee (co-payment), with public resources collected through general taxation. A national fund was established to provide the necessary resources to the regions to deliver the LEA. Any care provided above the LEA had to be funded through the regional budget. However, soon after the constitutional reform, some regions, due to weak managerial capacity and poorer government accountability, failed to reach the set goals, and the regional health budgets quickly ran into severe deficits. As a result, the central government had to adopt strict controls on regional healthcare spending to monitor and contain regional budgets. If the regional budget deficit exceeded 5% of total funding, regions formally committed themselves to designing an industrial reorganization programme and implementing a financial recovery plan, known as the *Piano di Rientro* (PdR) programme. Under the PdR, regions must identify the inefficient areas causing the deficits and take appropriate measures to recover from them. Within this framework, empirical evidence shows systematic regional heterogeneity both in the management of the budget and in the performance of the provision of public healthcare services. To account for these features of the Italian NHS, we include in the analysis the dummy variable ‘PdR’, which equals 1 if the region is subject to a financial recovery plan, zero otherwise, and the variable ‘LEA’ which measures regional degree of compliance with a nationally set target in terms of adequate level of public healthcare services. More explicitly, a region can be classified as: compliant (when the LEA score is > 160) or compliant with reserve (when the LEA score is > 130 but < 160) when public healthcare services are adequately provided, and critical (i.e., not compliant when the LEA score is < 130) when public healthcare services are not adequately provided.^22^

#### Variables measuring the quality of institutions

We use the IQI designed by Nifo and Vecchione [73] as a proxy for institutional quality at the local level. The structure of the IQI is inspired by the World Governance Indicator (WGI) proposed by Kaufmann et al. [57], and it is aligned with other initiatives for broader contexts, such as the European Quality of Government index (EQI) by Charron et al. [23]. It is based on data from ISTAT and other national research institutes and it is designed on five dimensions: (1) civic engagement, social cooperation, political participation and cultural liveliness (labelled Voice and Accountability), (2) quality of public services and policies in terms of public expenditure, waste management and environment policies formulated and implemented by the local government (labelled Government Effectiveness); (3) the rule of law measured in terms of crime against persons or property, magistrate productivity, trial times, tax evasion and shadow economy (labelled Rule of Law); (4) the degree of corruption as crimes committed against the Public Administration (labelled Control and Corruption) and (5) the ability of local government to promote policies fostering firms (labelled Regulatory Quality). The IQI is computed in such a way that higher values are associated with higher institutional quality.^23^

The overall IQI includes both elements of formal (from dimension two to five) and informal institutions (first dimension), as defined in Sect. Institutions and health: an overview.

Our choice of the IQI as an indicator of local institutional quality is corroborated by the economic literature that extensively employs it in various contexts of analysis [3, 33, 35, 38, 41],^24^ while the choice of ‘Government effectiveness’ and ‘Rule of law’ components stems from the intention to focus exclusively on two relevant aspects for population health:^25^ the ability of a government to guarantee the effectiveness of some public policies (public expenditure, waste management and environment policies) and the ability to provide an efficient legal system and compliance with the laws.

**Fig. 5:**
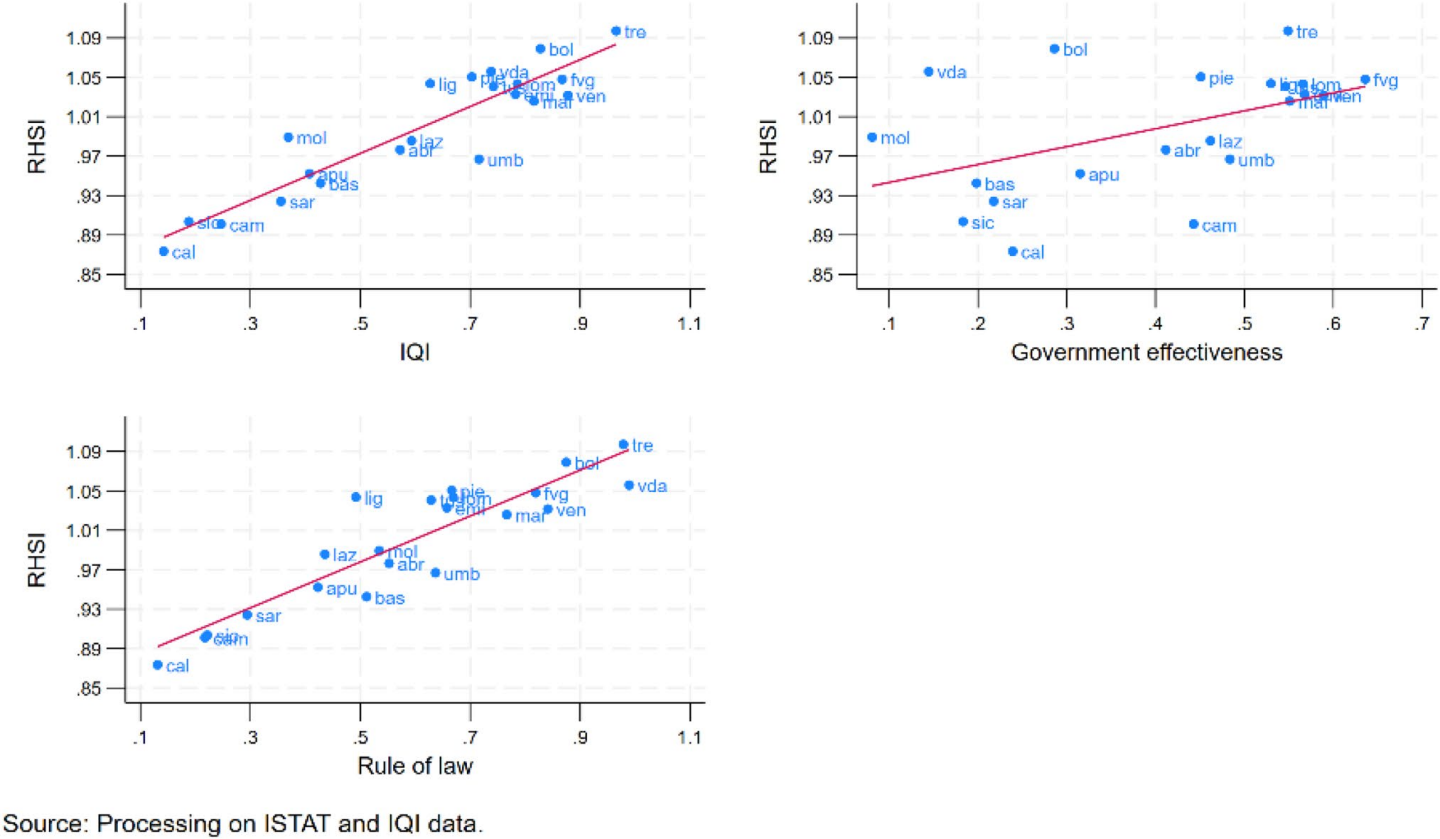
RHSI and Institutional Quality (average values 2011–2019)

Figure 5 shows that the RHSI is positively correlated to the IQI and the components ‘Government effectiveness’ and ‘Rule of law’. The Pearson correlation coefficients confirm the statistical significance of the correlations (Table 2). In particular, the data reveal values of correlation coefficients between the health status and IQI or its component ‘Rule of law’ higher than 0.8, while the correlation between RHSI and ‘Government effectiveness’ is much lower (0.4) but still significant.

**Table 2:** Pairwise correlation coefficients

	RHSI	IQI	Government effectiveness	Rule of law
RHSI	1.0000			
IQI	0.8448	1.0000		
	(0.00000)			
Government effectiveness	0.4334	0.6617	1.0000	
	(0.00000)	(0.00000)		
Rule of law	0.8285	0.9036	0.3363	1.0000
	(0.00000)	(0.00000)	(0.00000)	

#### Instrumental variables

The Instrumental Variable (IV) ‘Good transports’ is defined as the percentage of people declaring to be satisfied with public transports. This variable is created starting from the number of people who use public transports (bus/trolley bus/tram) and are very much and quite satisfied with: (1) timetables; (2) punctuality; (3) possibility of finding a seat; (4) speed of the bus; (5) cleanliness of the cars; (6) comfort of bus stops; (6) connectivity with other municipalities; (7) convenience of timetables; and (8) fares. These data are from ISTAT and are expressed as number of people (aged 14 and over) among 100 people with the same characteristics (basically percentages) at regional level. We obtain the IV ‘Good transports’ by computing the unweighted arithmetic mean of the quantities labelled with numbers (1) to (8).

The other IV ‘Clean streets’ is the percentage of households declaring to live in an area where streets are very much or quite clean. These data are from ISTAT and are expressed as number of households among 100 households with the same characteristics (basically percentages) at regional level.^26^

Several reasons address our interest towards these variables. First of all, they are widely used in literature as proxies for the efficiency and the effectiveness of the public services [19, 27] testing, in an indirect way, the quality of the formal institutions governing them. Secondly they are also correlated to the civic engagement of citizens (especially for the ‘Clean streets’ variable) representing a component of informal institutions.

The validity and power of these instruments is proved in Sect. Empirical results in which we report and discuss the Angrist–Pischke test of excluded instruments, the underidentification and the weak identification tests the weak-instrument-robust inference tests and the Hansen J statistic for overidentification of all instruments.

### Descriptive statistics

The descriptive statistics are reported in Table 3. On average, the RHSI is rather stable over time, as already discussed above with the support of graphs (see Figs. 2 and 3). Regarding the variables of the regional context, on average, per capita gross disposable income is almost 18,000 euros, with northern regions richer (20,000 euros) than southern regions (14,000 euros). On average, 60% of the individuals aged 25–64 have received at least a secondary school certification, with peaks of 65–70% in Lazio (centre) and Trento (north) but also very low percentages (less than 50%) in Apulia, Sicily and Sardinia (south). On average, 11 individuals out of 100 with the same characteristics are unemployed, with peaks of almost 23 in Calabria and Sicily (south). On average, income inequality, measured by the ratio between the income received by the richest 20% of the population and the income received by the poorest 20% of the population, is equal to 5, meaning that for every rich individual at the top of the income distribution, there are 5 poor individuals at the bottom of the distribution, with peaks of 10 in Campania and Sicily (south) and generally values higher than average in all southern regions. On average, 18 individuals out of 100 with the same characteristics may present drinking habits, with peaks of more than 20 individuals, especially in northern regions. Twenty-two percent of the total population is aged 65 + , with Liguria (north) being the oldest region: on average, almost 28% of the total population is classified as old. Regarding healthcare system variables, on average per 10,000 inhabitants, there are 95 doctors and dentists, nursing staff, technical health personnel and rehabilitation staff employed in public healthcare facilities and almost 33 hospital beds. Between 2011 and 2016, eight regions were subject to a financial restructuring programme (PdR), namely, Abruzzo, Apulia, Calabria, Campania, Lazio, Molise, Piedmont and Sicily, while from 2017 onwards, Piedmont exited the programme, having successfully recovered. Despite being on average fully compliant with LEA (173 scores), compliance is not stable over time, and many regions become complaint with reserve or critical over the period of 2011–2019, as also proven by the high standard deviation (30). Regarding quality of institutions variables, perception concerning law enforcement is generally higher than endowment of social and economic structures and of the administrative capability of regional governments in terms of health policies, waste management and environment (0.588 vs. 0.402), while overall quality of public institutions at the local level (including elements of formal and informal institutions) is higher (0.607). Finally, we report descriptive statistics of IVs: on average, around 57 out of 100 people with the same characteristics are satisfied with public transport, while more than 70 out of 100 households with the same characteristics declare to live in areas where the streets are clean.

**Table 3:** Summary statistics. Italy, 2011–2019

Variable	Mean	Std. Dev	Min	Max	N
RHSI	0.998	0.067	0.843	1.125	189
Socioeconomic component					
Income	17,935	3630	11,997	26,852	189
Education	60.283	6.768	46.300	71.100	189
Unemployment	11.136	5.248	2.890	23.420	189
Income inequality	5.216	1.254	3.300	10.000	189
Drinking habits	18.191	3.989	9.200	29.500	189
Old population	22.410	2.445	16.289	28.654	189
Healthcare system variables					
Staff	95.220	22.183	53.620	168.250	189
Beds	32.704	4.035	20.870	42.660	189
PdR	0.365	0.483	0	1	189
LEA	173.333	30.139	101	222	189
Quality of institutions variables					
IQI	0.607	0.242	0.072	1	189
Government effectiveness	0.402	0.178	0	0.690	189
Rule of law	0.588	0.242	0.068	1	189
Instrumental variables					
Good transports	56.570	14.742	19.6	87.61	189
Clean streets	73.228	9.366	45.7	90.4	189

## Empirical strategy

Since multidimensional and socioeconomic indices, based on social and economic determinants, should be used in ecological setting [4], we use the principal component analysis (PCA), as suggested by Friesen et al. [45], applied to our variables of regional context also to reduce data dimensionality and overcome potential multicollinearity issues.

The basic idea of PCA is to describe the variation of a multivariate dataset through uncorrelated linear combinations of the original variables. It is a technique that reduces the number of variables involved in the analysis and is thus widely used to summarize multiple indicators of socioeconomic contexts. Generally, the first few components represent most of the variation in the original dataset. We include in our empirical analysis the first principal component -the only one with an eigenvalue greater than one (3.8)- explaining around 65% of the variation of the six original variables. It can be interpreted as a proxy of socioeconomic well-being. We also ran the Kaise-Meyer-Olkin (KMO) test to check the overall consistency of the PCA in representing our data. The test gives an overall KMO value of 0.79 with partial values for each variable greater than 0.77. Given the overall PCA validation threshold of 0.6 [55], we conclude that the method can be applied to the original data without information loss. Detailed results on PCA are reported in Appendix B.

The fitted scores of the first component of the PCA, are then used in the second part of the empirical analysis in which we estimate the effects of institutions on the RHSI, controlling for healthcare system productive factors and policy variables.

The baseline model specification has the following form:

4 $${y}_{i,t}={\beta }_{0}+{\beta }_{1}{SE}_{it}+{\beta }_{2}{HS}_{it}+{\beta }_{3}{HP}_{it}+{\beta }_{4}{INST}_{it}+{\gamma }_{i}+{\varepsilon }_{it}$$

where ${y}_{i,t}$ is the RHSI observed in region *i* at year *t*, and ${\beta }_{1}$–${\beta }_{4}$ are our coefficients of interest that capture the effect of change in the explanatory variables on the health status for region *i* at year *t*. In particular, ${SE}_{it}$ refers to the socioeconomic component, measured by the fitted scores of the first component of the PCA; ${HS}_{it}$ refers to the healthcare system variables, i.e. personnel employed in public healthcare facilities and hospital beds; ${HP}_{it}$ refers to the healthcare system policy variables, i.e. PdR dummy and LEA score; and ${INST}_{it}$ represents the variables measuring the quality of the institutions, i.e. the IQI and its components ‘Government effectiveness’ and ‘Rule of law’. Finally, ${\gamma }_{i}$ is a discrete variable taking values 1 to 21 and identifying the regions, and ${\varepsilon }_{it}$ is the overall error term for region *i* at year *t*.

The empirical specification (4) is first estimated using the pooled OLS estimator with robust standard errors clustered at the regional level. However, due to the heteroskedasticity issue and to choose the most efficient estimation strategy, we perform both the Breusch‒Pagan Lagrange multiplier test and the Hausman test. In particular, the Hausman test reveals that the random effect estimation model can be considered appropriate ($Prob>{\chi }^{2}=0.5$). Random effects (RE) models, however, could suffer from cross-sectional dependence in the errors caused by possible common unobserved factors [32]. To overcome this problem, given that the number of groups (21) is greater than the number of time periods (9), we improve our empirical strategy by applying the panel-corrected standard error (PCSE) technique, which assumes heteroskedastic errors across groups [12].

However, as regional healthcare systems may be under greater pressure when the population is less healthy, leading to a deterioration in the quality of institutions, this potential reverse causal relationship between RHSI and IQI and its components may be a source of possible endogeneity.

We thus combine an IV approach with a two-way RE model to control for these potential problems and provide consistent estimates for Eq. (4). The IVs must be directly correlated with variables IQI and its components but uncorrelated with the dependent variable RHSI. The variables that we adopt, the percentage of people satisfied with local public transports (*GT*) and the percentage of families satisfied with cleanness of the streets (*CS*), have these characteristics. They indirectly measure the quality of institutions of the region -as the IQI (and its components) is conceived and calculated- through the ability of local authorities to efficiently and effectively meet the needs of the local population (*GT*) and also through the civic engagement of the citizens (*CS*). Formally, we estimate the following first-stage equation

5 $${INST}_{it}={\alpha }_{0}+{\alpha }_{1}{IV}_{it-1}+{\alpha }_{2}{Z}_{it}+{\varepsilon }_{it}$$

where *INST*_*i*,*t*_ represents the institutional variables, either IQI or its components ‘Government effectiveness’ and ‘Rule of law’, for region *i* at year *t*; *IV*_*i*,*t*−1_ represents the instrumental variables, either *CS* (when Eq. (5) refers to IQI, Column (13) of Table 4) or *CS* and *GT* (when Eq. (5) refers to ‘Government effectiveness’ and ‘Rule of law’, Column (14) of Table 4), for region *i* at year *t*−1;^27^*Z*_*i*,*t*_ refers to the set of area-level characteristics as discussed above; and ${\varepsilon }_{it}$ is the error term randomly distributed. We consider the lagged value of IVs to generate exogenous variation in variables IQI, ‘Government effectiveness’ and ‘Rule of law’ (*INST*). The validity and power of the instruments is tested through the following validation tests: the Angrist–Pischke test of excluded instruments, the under identification and the weak identification tests, with cutoffs provided by Stock and Yogo [87], the weak-instrument-robust inference tests and the Hansen J statistic for overidentification of all instruments.

**Table 4:** Empirical results, 2011–2019

	(1)	(2)	(3)	(4)	(5)	(6)	(7)	(8)	(9)	(10)	(11)	(12)	(13)		(14)	
	OLS	OLS	OLS	OLS	OLS	OLS	OLS	OLS	RE	RE	PCSE	PCSE	IV-RE		IV-RE	
Socioeconomic component																
SE	0.02868***	0.02313***	0.02144***	0.01657***	0.00256	0.00173	0.00002	0.00039	0.00464	0.00379	0.00256	0.00173	− 0.00685		− 0.00339	
	(0.00128)	(0.00210)	(0.00234)	(0.00281)	(0.00328)	(0.00301)	(0.00307)	(0.00295)	(0.00340)	(0.00327)	(0.00248)	(0.00253)	(0.00822)		(0.00714)	
Variables relative to the healthcare system resources																
Staff		0.00063***	0.00047**	0.00085***	0.00103***	0.00111***	0.00085***	0.00094***	0.00075***	0.00083***	0.00103***	0.00111***	0.00075**		0.00128***	
		(0.00021)	(0.00020)	(0.00022)	(0.00020)	(0.00020)	(0.00019)	(0.00020)	(0.00023)	(0.00025)	(0.00017)	(0.00018)	(0.00036)		(0.00032)	
Beds			0.00227**	0.00283***	0.00194**	0.00225**	0.00181**	0.00209**	0.00161**	0.00207***	0.00194**	0.00225***	0.00213**		0.00215**	
			(0.00106)	(0.00106)	(0.00092)	(0.00090)	(0.00088)	(0.00088)	(0.00071)	(0.00065)	(0.00084)	(0.00077)	(0.00094)		(0.00092)	
Policy variables																
PdR				0.01510***	0.02158***	0.02051***	0.01369**	0.01405**	0.01601**	0.01317**	0.02158***	0.02051***	0.02068*		0.01962*	
				(0.00543)	(0.00672)	(0.00646)	(0.00657)	(0.00658)	(0.00769)	(0.00595)	(0.00694)	(0.00677)	(0.01115)		(0.01157)	
LEA				0.00036***	0.00025**	0.00030***	0.00027***	0.00031***	0.00024*	0.00024**	0.00025**	0.00030***	0.00042**		0.00036*	
				(0.00011)	(0.00010)	(0.00011)	(0.00010)	(0.00011)	(0.00012)	(0.00011)	(0.00010)	(0.00012)	(0.00017)		(0.00020)	
Institutional variables																
IQI					0.14645***		0.10441***		0.13608***		0.14645***		0.25221***			
					(0.02147)		(0.02126)		(0.02977)		(0.01869)		(0.09484)			
Government effectiveness						0.06443***		0.04815***		0.06340***		0.06443***			0.15083**	
						(0.01751)		(0.01720)		(0.02413)		(0.01515)			(0.07236)	
Rule of law						0.12020***		0.08759***		0.11463***		0.12020***			0.12655***	
						(0.01759)		(0.01805)		(0.02668)		(0.01659)			(0.04621)	
Regional controls																
Regions							-0.00280***	-0.00227***								
							(0.00063)	(0.00064)								
Constant	0.99827***	0.93806***	0.87932***	0.75903***	0.69567***	0.66225***	0.77492***	0.73766***	0.74645***	0.71221***	0.69567***	0.66225***	0.62483***		0.60668***	
	(0.00261)	(0.01966)	(0.03480)	(0.04674)	(0.04231)	(0.04155)	(0.04557)	(0.04770)	(0.03810)	(0.03497)	(0.03750)	(0.04152)	(0.07960)		(0.07872)	
Observations	189	189	189	189	189	189	189	189	189	189	189	189	147		147	
R-squared	0.71108	0.72868	0.73799	0.75357	0.80235	0.81257	0.82072	0.82308	0.7976	0.8096	0.80235	0.81257	0.7577		0.7787	
Groups									21	21	21	21	21		21	
First-stage estimates of IQI (IV only)																
CS													0.00539***			
													(0.00123)			
First-stage estimates of Government effectiveness (IV only)																
CS															0.00639***	
															(0.00191)	
GT															0.00707**	
															(0.00169)	
First-stage estimates of Rule of law (IV only)																
CS															0.00579***	
															(0.00127)	
GT															0.00309**	
															(0.00112)	
F-stat. or Wald χ^2^	F(1, 187) = 500.26	F(2, 186) = 261.30	F(3, 185) = 192.73	F(5, 183) = 125.76	F(6, 182) = 148.88	F(7, 181) = 132.48	F(7, 181) = 138.92	F(8, 180) = 123.62	Wald chi2(6) = 542.43	Wald chi2(7) = 1124.38	Wald chi2(6) = 2893.20	Wald chi2(7) = 2225.32	Wald chi2(6) = 494.20		Wald chi2(7) = 599.18	
	Prob > F = 0.0000	Prob > F = 0.0000	Prob > F = 0.0000	Prob > F = 0.0000	Prob > F = 0.0000	Prob > F = 0.0000	Prob > F = 0.0000	Prob > F = 0.0000	Prob > chi2 = 0.0000	Prob > chi2 = 0.0000	Prob > chi2 = 0.0000	Prob > chi2 = 0.0000	Prob > chi2 = 0.0000		Prob > chi2 = 0.0000	
Angrist–Pischke test of excluded instruments																
IQI F statistic													8.22			
Prob > F													0.0095			
Government effectiveness F statistic															8.63	
Prob > F															0.0020	
Rule of law F statistic															9.76	
Prob > F															0.0011	
Kleibergen-Paap underidentification test																
LM statistic													6.27		7.06	
													(0.0123)		(0.0079)	
Wald statistic													9.00		18.95	
													(0.0027)		(0.0000)	
Kleibergen-Paap weak identification test (Wald F statistic)													8.22		8.59	
Weak-instrument-robust inference																
Anderson-Rubin Wald F test													4.92		3.2	
													(0.0383)		(0.0622)	
Anderson-Rubin Wald χ^2^ test													5.39		7.06	
													(0.0203)		(0.0293)	
Stock-Wright LM statistic													4.46		5.13	
													(0.0348)		(0.0769)	
Sargan-Hansen overidentification test of all instruments (J statistics)													0.000		0.000	

Robust Standard errors in parentheses. ***p < 0.01, **p < 0.05, *p < 0.1

The predicted values of the institutional variables (IQI, ‘Government effectiveness’ and ‘Rule of law’) derived from (5) ($\widehat{INST}$) are then used in the second-stage regression of the Eq. (4).

## Empirical results

We run our regressions on a balanced panel of 21 territorial units over the period of 2011–2019. The results are reported in Table 4, in which columns (1–8) present OLS estimation with controls introduced by blocks.

In Column (1), we simply focus on the effect of the socioeconomic component. The socioeconomic component is positively correlated with health status. This result is in line with the literature on the effect of socioeconomic status on health status [44].

In Columns (2) and (3), we add variables describing the national healthcare system. Both personnel employed in public healthcare facilities and ordinary hospital beds have a positive effect on RHSI, in line with the economic literature [10] modelling the healthcare system as a “production system” using capital (‘Beds’) and labour (‘Staff’) as inputs.

In Column (4), we also account for policy variables. The coefficient of the PdR variable is positive and significant. However, this finding requires a cautious interpretation. The empirical literature on this issue highlights that the impact of PdR programmes on citizens’ health is quite sensitive to the well-being or health indicator adopted, and some results appear to be contradictory [77]. In this complex framework, we are aware that our findings may be dependent on the composite health indicator used and that more in-depth analyses are needed to investigate in detail the impact of financial recovery plans on disaggregated health status measures.^28^ This is not, of course, the focus of our research, which aims to evaluate instead the relationship between institutional quality and health. Therefore, PdR variable is introduced as a mere control, and in our analysis, it captures the possible effect of rationing inefficient healthcare spending on the health status. The results on the LEA variable indicate that the more compliant the region is with the national healthcare targets, the more adequate the level of public healthcare services is and the higher the RHSI [21].

Finally, in Columns (5–8), we also introduce variables measuring the quality of the institutions. We first look at the effect of the overall quality index (IQI): the higher the quality of institutions is, the greater the effect on health status is. We then use IQI components as a robustness check of this positive correlation between quality of the institutions and health. In particular, we focus on the ‘Government effectiveness’ component, including the endowment of social and economic facilities in Italian regions and the administrative capability of regional governments in terms of health policies (the regional healthcare deficit), waste management and environment factors affecting health (i.e., the separate waste collection and the urban environment index including dimensions related to air quality, water quality, public parks etc.). We also consider the ‘Rule of law’ component containing elements of formal institutions (ruled by law and regulation) and summarizing data on crime, activities of the magistracy, tax evasion and submerged economy. In all cases, we find that the quality of the institutions is highly significant with a positive sign, meaning that the RHSI increases with increasing quality of regional institutions.

Our results confirm the first hypothesis set in Sect. Institutions and health: an overview (Hp1: At Italian regional level, higher institutional quality is associated with higher health status of the population.), even when we introduce a variable identifying the region: health worsens as we move from the north to the south of the country. Our results also confirm the second hypothesis (Hp2: Regional socioeconomic factors do not affect overall population health when local institutions are well-functioning and regions present a higher level of compliance with national standards in terms of public healthcare services.): when introducing institutional quality variables, the socioeconomic component loses significance. This is probably because when the regional provision of public health services corresponds to national standards and the institutions are well-functioning and guarantee their effectiveness, the health needs of the target population are better met, regardless of socioeconomic background.

Results are also confirmed using the RE estimator (Columns (9) and (10)) and the PCSE regression (Columns (11) and (12)).

The last two columns ((13) and (14)) show the estimations of the IVs approach. We only comment here the first-stage results and tests of the IV-RE model, reported at the bottom of Table 4, as second-stage results are similar to those reported in Columns (9) and (10).

We expect that an increase in the share of families declaring to live in areas where streets are clean (*CS*) and an increase in the percentage of people satisfied with public transport (*GT*) show a positive significant correlation with the quality of institutions (IQI, ‘Government effectiveness’ and ‘Rule of law’).

In the first stage all instruments are statistically significant and with the expected sign. The IV CS fall within the 1% confidence level, while the IV GT is within the 5% level.

The Angrist–Pischke test of excluded instruments is passed for all institutional variables meaning that the excluded instruments do not influence the dependent variable. Since the *p*-values of the Kleibergen-Paap test are very low (for both the Wald and LM statistics), the under-identification hypothesis is rejected: the model is identified. Besides being relevant, the instruments are also valid. The Sargan-Hansen test does not reject the null hypothesis that all instruments are exogenous and therefore the model is exactly identified. The strength of the instruments is verified through the F statistic of the Kleibergen-Paap weak identification test. Based on the critical values of Stock and Yogo [87],^29^ the F statistic relative to Column (13) with a value of 8.22 is between the critical values of 15% and 20% (closer to 15% value), while the F statistic relative to Column (14) with a value of 8.59 is over the critical values of 10%. We can conclude that the relevance of our IVs is good in Column (13) and very good in Column (14). Last, according to the Anderson-Rubin Wald F and *χ*^2^ tests and the Stock-Wright LM statistic, the hypothesis of joint significance of endogenous regressors is rejected at 5–10% level.

## Conclusions

This paper assesses the relationship between the health status of the Italian population measured at the regional level and quality of institutions.

Since in Europe health is a primary responsibility of the member states, not of the EU, which only provides a coordinated approach at both EU and global level, we present Italy as a case study to show the relevance of our hypothesis. We believe that the empirical setting proposed in our paper can be used to explain the role of institutions also in other federal/regional contexts characterised by unevenly distributed resources, inequalities, and non-homogeneous quality of institutions such as Austria, Germany, Belgium and/or by sub-national level of health management such as Finland, Spain, Sweden. Moreover, the issue of institutional quality and health outcomes might be particularly relevant for the international socio-political debate in which the question of the variables affecting health and possible policy measures are a growing concern, also considering the Sustainable Development Goal 3 of the 2030 Agenda (Ensure healthy lives and promoting well-being for all at all ages).

The analysis departs from the construction of a multidimensional composite indicator of the health status, built on the combination of elements relating to both objective measures of health status and self-reported health, and then moves to investigating the role of socioeconomic factors, healthcare facilities, health policies and quality of institutions on such multidimensional indicator.

Our analysis shows that institutional quality is an important driver of population health status in the Italian regional context. In particular, the analysis points out that when local institutions are well-functioning and regions present a higher level of compliance with national standards in terms of public healthcare services, the role of socioeconomic factors becomes less important. In other words, when institutions are efficient and effective and the regional healthcare services provision is coherent with national guidelines, then socioeconomic differences become secondary drivers for health.

Our findings suggest that more effort should be made to increase the quality of local institutions in regions where this is lower. Such an increase can be achieved, for example, by investing in the quality of human capital engaged in local public administrations and by training and focusing on more educated and skilled personnel.

Policymakers should therefore rethink the institutional agenda on health disparities and set investments in intersectionality, i.e., on an integrated ground to consider the multiple factors involved in shaping health conditions, from individual socioeconomic characteristics and social position to the role of institutions. Thus, investing in the quality of regional institutions and ensuring regional compliance with the national targets could be a further policy instrument to fight inequalities from a different angle.

Our analysis could be further delved into, and it calls for future research. A possible extension of the analysis concerns the investigation of the role of public institutions in the COVID-19 era with the purpose of understanding whether the pandemic has affected the relationship between health status and institutional quality.

## Appendix A

See Table 5.

**Table 5:** The Regional Health Status Indicator

Territorial units	2011	2012	2013	2014	2015	2016	2017	2018	2019
Abruzzo	1.006005	0.950131	0.979884	0.978552	0.980051	0.923919	0.998027	0.991702	0.979796
Aosta Valley	1.043417	1.090181	1.039776	1.0684	0.98404	1.074189	1.092846	1.059099	1.050998
Apulia	0.970197	0.936914	0.90272	0.935695	0.94592	0.950524	0.999652	0.961395	0.967183
Basilicata	0.977652	0.914295	0.942052	1.002537	0.981851	0.959042	0.893928	0.896916	0.915159
Bozen	1.063425	1.10081	1.124627	1.017253	1.105604	1.056364	1.071146	1.100266	1.071348
Calabria	0.857147	0.895282	0.852978	0.844219	0.870336	0.893398	0.842574	0.898872	0.906546
Campania	0.910374	0.888969	0.863519	0.884589	0.882956	0.934216	0.889736	0.906014	0.949364
Emilia Romagna	1.011117	0.987525	1.071185	1.077238	1.027668	1.044915	1.045494	1.01329	1.016205
Friuli-Venezia Giulia	1.044687	1.034436	1.070017	1.031658	1.045374	1.086664	1.028331	1.046958	1.044456
Lazio	0.975377	1.017584	0.985693	0.977511	0.976022	0.968883	0.966238	1.005906	0.997487
Liguria	1.071257	1.022907	1.053026	1.057235	1.027761	1.075003	1.039664	1.020184	1.025708
Lombardy	1.041559	1.019822	1.053778	1.034264	1.068435	1.062805	1.030907	1.039183	1.038692
Marche	1.052937	1.045407	1.034203	1.008148	0.984154	1.02912	1.053748	1.016513	1.008575
Molise	0.93646	1.012345	0.950161	1.059637	0.96597	0.973999	1.021651	1.004268	0.978884
Piedmont	1.054812	1.042837	1.028789	1.064953	1.060188	1.053244	1.043885	1.058162	1.046867
Sardinia	0.908531	0.941005	0.961219	0.846494	0.950659	0.887887	0.949152	0.926301	0.94577
Sicily	0.908313	0.916135	0.921131	0.906845	0.88776	0.889085	0.87745	0.930262	0.895401
Trento	1.101547	1.079767	1.077278	1.122575	1.107094	1.088249	1.111258	1.090465	1.095825
Tuscany	1.005993	1.034138	1.058599	1.039258	1.073954	1.037965	1.052067	1.04423	1.019615
Umbria	0.977734	0.994523	0.978542	0.98473	0.995928	0.942398	0.932109	0.91122	0.985012
Veneto	1.048408	1.04476	1.009276	1.010603	1.041737	1.030136	1.017096	1.043208	1.039097

Source: our elaboration on ISTAT data

## Appendix B Principal Component Analysis (PCA)

Table 6 reports the principal component or correlations between the variables we use to characterize the socioeconomic context. The eigenvalues of the correlation matrix, ordered from largest to smallest, add up to the sum of the variances of the variables in the analysis (the “total variance” of the variables). Because we are analysing a correlation matrix, the variables are standardized to have unit variance, so the total variance is 6 (the maximum number of components). The eigenvalues are the variances of the principal components. The first principal component has variance 3.84, explaining 64% (3.84/6) of the total variance. The second principal component has variance 0.97 or 16% (0.97/6) of the total variance. And so on. As almost 65% of the variance (last column) is contained in the first principal component (labelled Component 1), we just list Component 1 in Table 7. As Component 1 does not contain all information in the data, some of the variances in the variables are unaccounted for or unexplained. These equal the sums of squares of the loadings in the deleted components, weighted by the associated eigenvalues. The average unexplained variance is equal to the overall unexplained variance of 36% (= 1–0.64, where 0.64 is the explained variance of Component 1). As expected, component 1 has positive loadings on income, education and the elderly population (that in Italy holds a larger share of wealth compared to the young population, which gives the elderly a more stable socioeconomic condition), and negative loadings on income inequality and unemployment. Results on drinking habits might seem misleading. However, as specified by ISTAT [52], the ‘consumers at risk’ for drinking habits are identified as “individuals who have at least one risk behaviour, exceeding the daily consumption of alcohol (according to specific thresholds for sex and age) or concentrating on a single occasion of consumption the intake of 6 or more units of any alcoholic drink (binge drinking)”. This style of drinking overlaps somewhat with social drinking, which potentially captures aggregate social behaviour, especially among youth, as evidenced in the sociological literature that identifies drinking as a socially-based leisure activity [89, 92]. Table 8 reports the Kaiser–Meyer–Olkin (KMO) test to assess validity of the PCA and check its overall consistency in representing our data. The test gives an overall KMO value of almost 0.80 with partial values for each variable greater than 0.76. Given the overall PCA validation threshold of 0.6 [55], we conclude that the method can be applied to the original data without information loss.

**Table 6:** Principal components/correlation

Component	Eigenvalue	Difference	Proportion	Cumulative
Component 1	3.84403	2.86655	0.6407	0.6407
Component 2	0.977482	0.384145	0.1629	0.8036
Component 3	0.593337	0.263475	0.0989	0.9025
Component 4	0.329862	0.157043	0.0550	0.9575
Component 5	0.17282	0.0903491	0.0288	0.9863
Component 6	0.0824704		0.0137	1.0000

**Table 7:** Eigenvectors of Principal Component 1

Variable	Component 1
Income	0.4597
Education	0.3968
Unemployment	− 0.4856
Income inequality	− 0.4239
Drinking habits	0.3819
Old population	0.2644

**Table 8:** Kaiser–Meyer–Olkin test

Variable	KMO
Income	0.8137
Education	0.7738
Unemployment	0.7608
Income inequality	0.8458
Drinking habits	0.7701
Old population	0.8319
Overall	0.7937

## Footnotes

1 For sake of simplicity, throughout the paper we use the terms “region” or “regional” to address all the territorial units, including the two provinces.

2 North points out that “*the difference between formal and informal rules is one of degree…. formal rules can complement and increase the effectiveness of informal constraints. They may lower information, monitoring and enforcement costs and hence make informal constraints possible solutions to more complex exchange*” [74] pp.46–47).

3 Studies on informal institutions often adopt different interpretations or refer to a few typologies. For an overview on the main types of informal institutions see Lauth [65].

4 Through the whole text, we have used the term ‘health’ to refer to the state of complete well-being, the term ‘healthcare’ (replaceable with health care) to refer to the improvement of health via the prevention, diagnosis, treatment, cure of disease, illness, injury, and the term ‘healthcare system’ to refer to an organization of people, institutions and resources that delivers healthcare services and that arranges for their financing to meet the health needs of target populations (for example, the National Health Service).

5 More extensively, the authors identify four mechanisms of connecting welfare state and health distribution: redistribution, compression, mediation and imbrication. For a detailed analysis, see Beckfield et al. [14].

6 Decommodification refers to “the extent to which individuals and families can maintain a normal and socially acceptable standard of living regardless of market performance” [39], p. 86).

7 An earlier version of the indicator can be found in Antonelli and Marini [6].

8 Self-reported health is a health measure based on survey questions. Although the literature has pointed out that it might be affected by self-reporting bias (see, for example, [9]), it remains one of the most popular ways to measure health.

9 For a detailed description of these variables, please check the online help of the operating system Health for All-Italia managed by the Italian Institute of Statistics (ISTAT) and its data warehouse (http://dati.istat.it/).

10 The 16 causes of death are: AIDS; circulatory system diseases; digestive system diseases; diseases of the endocrine glands, nutrition and metabolism; diseases of the genitourinary system; complications in pregnancy, childbirth and the puerperium (age 15-49); infectious diseases; diseases of the muscular system and connective tissue; diseases of the nervous system and sense organs; diseases of the skin and subcutaneous tissue; psychic disorders; respiratory system diseases; diseases of the blood and haematopoietic organs and immune disorders; trauma and poisoning; cancer; and other causes.

11 See https://hdr.undp.org/data-center/human-development-index#/indicies/HDI

12 See https://www.weforum.org/reports/the-global-competitiveness-report-2020

13 On this point see also Di Bella et al. [36].

14 The geometric mean is recognized as a more reliable measure to summarize indicators than the arithmetic mean being more robust against outliers [36]. Each domain has equal weighting for the final indicator as in the methodology used by Afonso et al. [2].

15 The ‘Well-Being and Sustainability’ (*Benessere Equo e Sostenibile*, BES) project aims at evaluating the progress of society considering a wide set of indicators. For details see: https://www.istat.it/en/well-being-and-sustainability

16 More information is available here: https://sites.google.com/site/institutionalqualityindex/home

17 See, for example, Beckfield et al. [13, 14] and the other references cited in the Introduction.

18 Pickett and Wilkinson [78] use income inequality as a macro-economic measure of the scale of differentiation of social status to test the effects of a more unequal society on a multidimensional well-being indicator.

19 Our choice is fundamentally linked to the Italian context, where the elderly holds a larger share of wealth than the young population, which gives the elderly a more stable socioeconomic condition [11].

20 Drinking habits could represent a proxy for sociality as, according to ISTAT [52], such habits are defined as a daily overconsumption of drinks or the so called “binge drinking” often associated to aggregate social behaviour, especially among young people, as highlighted by sociological literature [89, 92].

21 The Italian NHS was founded in 1978 and was based on the principle of universal coverage. It was financed mainly through general taxation and resources were allocated to the regions according to a capitation system.

22 More information available here: https://www.salute.gov.it/portale/lea/menuContenutoLea.jsp?lingua=italiano&area=Lea&menu=leaEssn

23 Further details on the items of the IQI and the procedure of calculation is available on. https://sites.google.com/site/institutionalqualityindex/home?pli=1

24 Regarding the health sector, De Luca et al. [33] provide a regional analysis investigating the effect of the institutional quality, as measured by the IQI, on Caesarean section rates (used as a measure of healthcare inappropriateness) and on health outcomes, as measured by reductions in heart attack, hip fracture and stroke mortality rates.

25 See Sect. Institutions and health: an overview for literature references.

26 The original ISTAT data concern “dirty streets”. We then take the complementary percentage and obtain the IV ‘Clean streets’ used in our analysis.

27 When we replace IQI with its two components, in the first-stage we have to instrument two variables (‘Government effectiveness’ and ‘Rule of law’) and therefore we need two instruments.

28 For a more specific analysis on this argument see Cirulli and Marini [27].

29 Stock-Yogo weak ID test critical values for F(1, 20) are 16.38 (10% maximal IV size), 8.96 (15% maximal IV size), 6.66 (20% maximal IV size) and 5.53 (25% maximal IV size), while those for F(2, 20) are 7.03 (10% maximal IV size), 4.58 (15% maximal IV size), 3.95 (20% maximal IV size) and 3.63 (25% maximal IV size).
